# On Unavoidable Hemorrhage

**Published:** 1845-06

**Authors:** 

**Affiliations:** The University of Edinburgh


*On unavoidable Hemorrhage.* By PROFESSOR SIMPSON, of the University of Edinburg.—From a long inquiry, the following deductions are made by Professor Simpson.

Our inquiry, as far as we have hitherto proceeded, seems legitimately to admit of the following deductions.

1. The complete separation and expulsion of the child, in cases of unavoidable hemorrhage, is not so rare an occurrence as accoucheurs appear generally to believe.

2. It is not by any means so serious and dangerous a complication as might *a priori* be supposed.

3. In nineteen out of twenty cases in which it has happened, the attendant hemorrhage has either been at once altogether arrested, or it has become so much diminished as not to be afterwards alarming.

4. The presence or absence of flooding after the complete separation of the placenta, does not seem in any degree to be regulated by the duration of time intervening between the detachment of the placenta and the birth of the child.

5. In ten out of one hundred and forty-one cases, or in one out of fourteen, the mother died after the complete expulsion or extraction of the placenta before the child.

6. In seven or eight out of these ten casualties, the death of the mother seemed to have no connection with the complete detachment of the placenta, or with results arising directly from it, and if we do admit the three remaining cases, (which are doubtful), as leading by this complication to a fatal termination, they would still only constitute a mortality from this complication, of three in one hundred and forty-one, or about one in forty-seven cases.

7. On the other hand, under the present established rules of practice, one hundred and thirty-four mothers died in three hundred and ninety-nine placental presentations, or about one in three.—*London and Edinburgh Monthly Journal.*

